# Supplementary material for: Bombardier beetles repel invasive bullfrogs
Source: PeerJ. 2022 Sep 15;10:e13805. doi: 10.7717/peerj.13805 (PMC9482772; doi:10.7717/peerj.13805)
Supplement: Supplemental Information 3 — (A) Study region (open rectangle). (B) Bullfrog-invaded (red circles, n = 4) and non-invaded sites (black circles, n = 2). White and blue areas represent land and sea (or lakes), respectively. The maps were modified from the Geospatial Information Authority of Japan (http://maps.gsi.go.jp/). [file peerj-10-13805-s003.pdf]

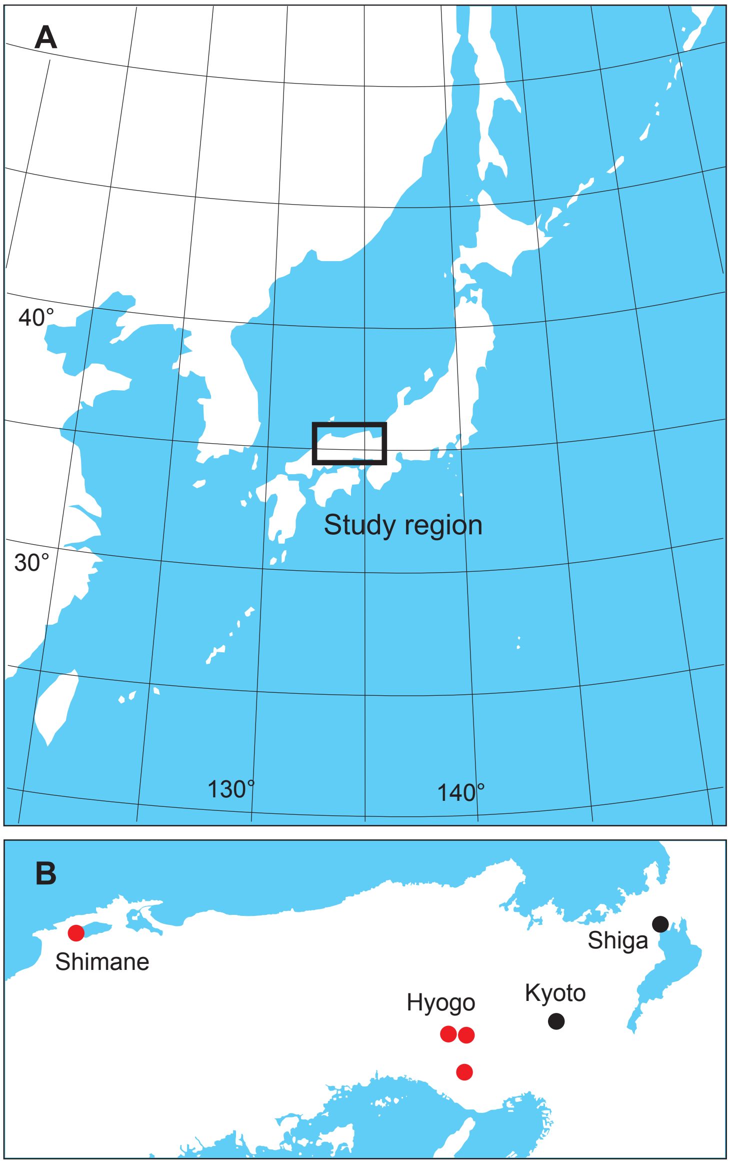

Figure S1 Study region and sites. (A) Study region (open rectangle). (B) Bullfrog-invaded (red circles, $n = 4$) and non-invaded sites (black circles, $n = 2$). White and blue areas represent land and sea (or lakes), respectively. The maps were modified from the Geospatial Information Authority of Japan (http://maps.gsi.go.jp/).
